# Novel Strategy for Surface Modification of Titanium Implants towards the Improvement of Osseointegration Property and Antibiotic Local Delivery

**DOI:** 10.3390/ma16072755

**Published:** 2023-03-29

**Authors:** Isabela Rocha da Silva, Aline Tavares da Silva Barreto, Renata Santos Seixas, Paula Nunes Guimarães Paes, Juliana do Nascimento Lunz, Rossana Mara da Silva Moreira Thiré, Paula Mendes Jardim

**Affiliations:** 1 COPPE/Program of Metallurgical and Materials Engineering (PEMM), Universidade Federal do Rio de Janeiro (UFRJ), Rio de Janeiro 21941-598, RJ, Brazil; 2 Graduation Program in Nanobiosystems, Universidade Federal do Rio de Janeiro (UFRJ), Duque de Caxias 25240-005, RJ, Brazil; 3 Faculdade de Odontologia, Universidade do Estado do Rio de Janeiro (UERJ), Rio de Janeiro 20551-030, RJ, Brazil; 4 Divisão de Metrologia de Materiais, Instituto Nacional de Metrologia, Qualidade e Tecnologia (Inmetro), Xerem 25250-020, RJ, Brazil

**Keywords:** titanium, hydrothermal treatment, surface modification, local drug delivery system, osseointegration

## Abstract

The topography and chemical composition modification of titanium (Ti) implants play a decisive role in improving biocompatibility and bioactivity, accelerating osseointegration, and, thus, determining clinical success. In spite of the development of surface modification strategies, bacterial contamination is a common cause of failure. The use of systemic antibiotic therapy does not guarantee action at the contaminated site. In this work, we proposed a surface treatment for Ti implants that aim to improve their osseointegration and reduce bacterial colonization in surgery sites due to the local release of antibiotic. The Ti discs were hydrothermally treated with 3M NaOH solution to form a nanostructured layer of titanate on the Ti surface. Metronidazole was impregnated on these nanostructured surfaces to enable its local release. The samples were coated with poly(vinyl alcohol)—PVA films with different thickness to evaluate a possible control of drug release. Gamma irradiation was used to crosslink the polymer chains to achieve hydrogel layer formation and to sterilize the samples. The samples were characterized by XRD, SEM, FTIR, contact angle measurements, “in vitro” bioactivity, and drug release analysis. The alkaline hydrothermal treatment successfully produced intertwined, web-like nanostructures on the Ti surface, providing wettability and bioactivity to the Ti samples (Ti + TTNT samples). Metronidazole was successfully loaded and released from the Ti + TTNT samples coated or not with PVA. Although the polymeric film acted as a physical barrier to drug delivery, all groups reached the minimum inhibitory concentration for anaerobic bacteria. Thus, the surface modification method presented is a potential approach to improve the osseointegration of Ti implants and to associate local drug delivery with dental implants, preventing early infections and bone failure.

## 1. Introduction

Titanium implants are a safe and predictable alternative for rehabilitation treatments in dental surgeries, and their success is related to osseointegration. Titanium is considered the material of choice for the manufacture of implants due to its mechanical properties, chemical stability, and biocompatibility [1,2]. Surface treatments such as alkaline hydrothermal treatment have been developed and improved to modify and optimize the physical–chemical properties of the surface, such as roughness, topography, wettability, and its composition, influencing cellular events during the healing process [3,4]. Such treatment enables the growing of a layer of titanate nanostructures on the Ti surface, increasing its roughness, and consequently its surface area, and hydrophilicity, favoring protein adsorption and cell adhesion and proliferation [4]. The morphology of nanostructures is directly dependent on the experimental conditions used for hydrothermal treatment.

Infections associated with the surgical procedures during dental implantation are one of the causes of osseointegration failure, leading to its loss [5]. Such infections can be caused by contamination of bacteria that already exist at the surgical site, especially in patients with pre-existing periodontal disease [6]. The most critical moment regarding early loss induced by infection is when the implant comes into contact with human cells and local microorganisms in the initial moments after its installation [7]. Prophylactic antibiotic therapy has been proposed to decrease bacterial levels at the site to be instrumented. The pre-established biofilm restricts the diffusion of molecules and bacterial sensitivity to antibiotics, meaning that the systemic prophylactic antibiotic therapy will not have the desired effectiveness [8]. Therefore, it is desirable to develop strategies with a local antibiotics release approach as an alternative to systemic oral antimicrobial therapy [9]. The use of biodegradable material in combination with titanium implants as a local drug delivery system is an interesting approach. 

Hydrogels have gained considerable attention in recent years for their unique physical–chemical properties, which are similar to those of living tissues and include high water content, soft and rubbery consistency, and low interfacial tension with water or biological fluids [10]. These materials can be defined as a 3D network formed from physical or chemical crosslinked hydrophilic polymers. They can swell in contact with water or biological fluids without losing their structural integrity [11]. Poly(vinyl alcohol)—PVA is a biodegradable, non-toxic, water-soluble, and hydrogel-forming polymer [12]. Due to its particular properties, such as chemical versatility, great biocompatibility to different cell lines, and high swelling capacity, PVA hydrogel has been recognized as potential biomaterial for various pharmaceuticals and biomedical applications, including as a matrix for drug delivery systems and as scaffolds for bone tissue engineering. Furthermore, PVA can only be solubilized in pure water, without needing any acid or alkaline solution, which could be a potential risk for cells [13,14]. 

Metronidazole is an antibacterial compound belonging to the class of imidazoles, a subgroup of nitroimidazoles, which acts against a wide variety of microorganisms [15]. It is clinically effective in the treatment of periodontitis and peri-implantitis caused by anaerobic bacteria [16]. It presents a good therapeutic response, but its high toxicity after continuous systemic doses can, for example, manifest gastrointestinal disorders, dizziness, and headaches, among others, often causing the patient to discontinue treatment [15]. Thus, the local delivery of metronidazole could reduce the systemic toxicity of the antibiotic and increase its efficiency even with lower doses. 

In the present work, we propose a surface treatment methodology to titanium implants aiming to provide antimicrobial properties by the local release of metronidazole and the improvement of the osseointegration capacity obtained by the incorporation of nanostructures on the titanium surface. The proposed system consists of a Grade 4 Ti sample functionalized with a nanostructured titanate layer produced by hydrothermal treatment, impregnated with metronidazole and coated with PVA hydrogel to control antibiotic release. Grade 4 Ti has been the industry standard for dental implants for years due to its high strength and low malleability. The great advantage of surface modification is that the external layers of the materials can be modified without affecting its volumetric characteristics, such as mechanical properties. In other words, it is possible to change biomaterial–cell interaction without changing physical and chemical bulk properties.

## 2. Materials and Methods

### 2.1. Preparation of Titanate Nanostructured Coating

Grade 4 Ti circular cross-section bars (Titanews Industria e Comercio de Titanio LTDA, Barueri, Brazil) were used in the alkaline hydrothermal treatment as a surface substrate for the growth of the titanate layer. Before treatment, the Ti bars were cut in a cutting machine (ISOMET 4000 model, Buehler), forming discs 12 mm in diameter and 2 mm in thickness, which were mechanically polished with abrasive silicon carbide sandpaper of different grades (220, 400, 600, and 1500) using a metallographic polisher/sander machine (Aropol 2V—Arotec, Cotia, Brazil), followed by ultrasonic cleaning for 10 min with acetone, ethanol, and distilled water, sequentially. The alkaline hydrothermal treatment was carried out in a high-pressure reactor (BR-500–Berghof, Eningen, Germany) under 3M NaOH solution at 150 °C for 6 h. After the synthesis reaction, the samples were washed by immersion in distilled water four times for 10 min, as will be described below.

### 2.2. Loading of Metronidazole in Nanostructures

Metronidazole (MNZ) (0.5% (*m*/*v*) metronidazole injection solution, Fresenius Kabi Brasil Ltd. (Mount Kuring-gai, NSW, Australia) was added to the coated Ti discs to interact with the nanostructures and to compose the drug delivery system. The MNZ concentration was selected based on the Minimum Inhibitory Concentration to eliminate anaerobic bacteria, on the concentration range that could avoid cytotoxicity, and on the minimum concentration that could be read by a UV-Vis spectrophotometer. Firstly, a 25 μL-drop of MNZ was pipetted on to the discs and allowed to dry at room temperature. After drying, another drop was added. This procedure was repeated eight times until 22.2 µg/mL of MNZ had been deposited. In the case of systems with a thicker layer of PVA coating, it was necessary to deposit a higher volume of MNZ (33.3 µg/mL) in order to maintain the released amount of MNZ within the range of the sensitivity of the method. 

### 2.3. Polymeric Coating of Nanostructured Samples 

Poly(vinyl alcohol) (PVA) (Mw 85.000–124.000 g/mol, degree of hydrolysis > 99%, Sigma–Aldrich, Saint Louis, MO, USA) was used to coat the MNZ-loaded samples in order to control the drug release. Two different groups were produced by this procedure for comparative purposes, a group with one layer and a group with six layers of polymeric coating. For the production of one PVA layer, the PVA was dissolved in distilled water at 90 ± 2 °C for 4 h under magnetic stirring to obtain 10% (*m*/*v*) PVA aqueous solution. Then, the solution was cooled under stirring to 50 °C, and 0.2 mL of PVA solution was pipetted onto the samples. The spin coating technique was performed at a rotational speed of 400 rpm for 10 s, followed by a rotational speed of 4000 rpm for 60 s, to ensure even coverage. In the case of the group with six layers, this procedure was repeated six times. After the coating procedure, all the samples were left in a ventilated oven for 20 h at 50 °C.

### 2.4. Crosslinking of Polymeric Coating

In order to crosslink the polymeric films, the PVA-coated samples (TTNT + MNZ + 1PVA, TTNT + MNZ + 6PVA groups) were irradiated with gamma rays of Cobalt-60 at a dose of 25 KGy. By using this procedure, the samples could be sterilized concomitantly with PVA chain crosslinking [17]. The experimental groups were named as shown in Table 1.

### 2.5. Microstructural Characterization

After hydrothermal treatment, the samples were analyzed by Grazing Incidence X-ray diffraction (GIXRD) and Scanning Electron Microscopy (SEM). The GIXRD (X’pert PRO/PANalytical, Malvern, UK) patterns were collected using CuKα radiation (λ = 1.5418 Å) and the incidence angle of the beam in relation to the samples’ surface of 1°. The surface morphology of the samples was examined by SEM using a Versa 3D microscope (Thermo Fisher, Helios/Thermo Fisher, Waltham, MA, USA) operating at an accelerating voltage of 20 kV. The samples were gold-coated prior to the analysis. The chemical bonds of the samples were analyzed by a FTIR spectrometer (Nicolet 6700, Thermo Scientific, Waltham, MA, USA) equipped with an ATR (Attenuated Total Reflection) accessory and using a resolution of 4 cm^−1^ in the region of 4000–650 cm^−1^, with an average of 32 scans. The signal of the obtained spectra was processed with the Origin Pro version 9.1 software using the Savitzky–Golay algorithm (five smoothing points) and normalized to [0, 1].

### 2.6. Apatite Deposition—“In Vitro” Bioactivity Assay

The samples were soaked in simulated body fluid (SBF) to assess their bioactivity by examining the formation of calcium phosphate crystals on the surface. SBF was prepared according to Kokubo’s formulation, and the bioactivity test was performed in accordance with ISO/FDIS 23317 (Implants for surgery—In vitro evaluation for apatite-forming ability of implant materials). The alkaline hydrothermally treated Ti samples and the untreated Ti (control sample) were soaked in SBF at pH = 7.4 and a constant temperature of 36.5 °C [18] for 14 days. After the soaking period, the samples were gently washed with distilled water and dried in a desiccator at room temperature. The formation of calcium phosphate crystals after the soaking period was investigated by means of SEM (Vega/Tescan operating at an acceleration voltage of 20 kV) and GIXRD (X’pert PRO/PANalytical, Malvern, UK). The samples were gold-coated prior to SEM analysis.

### 2.7. “In Vitro” Cytotoxicity Assay

To evaluate the influence of the hydrothermal treatment on the samples, a standardized cytocompatibility assay was performed (ISO 10993-5:2009—Biological evaluation of medical devices—Part 5: Tests for in vitro cytotoxicity). The 3-(4,5-dimethylthiazol-2-yl)-2,5-diphenyltetrazolium bromide (MTT) assay was performed with MG-63 cells (human fibroblast from osteosarcoma, Code 0173, Cell Bank of Rio de Janeiro, Brazil). 

Ti and Ti + TTNT discs were separately soaked in no supplemented DMEM for 24 h to produce the extracts. Concomitantly, cells were seeded in a 96-well plate at a density of 2 × 10^4^ cells/well and incubated in 200 µL of high-glucose Dulbecco’s modified Eagle’s medium GlutaMAX (hgDMEM) supplemented with 10% *v*/*v* of fetal bovine serum (FBS), penicillin (100 U·mL^−1^)/streptomycin (100 µg·mL^−1^) (all Gibco Biosciences, Waltham, MA, USA), and 2.5µg·mL^−1^ amphotericin B (Sigma–Aldrich, Saint Louis, MO, USA)—XPAN mediun—at 37 °C, in an atmosphere of 5% CO_2_. After overnight incubation (approximately 24 h), the EXPAN medium was replaced by the extracts, and the cells were incubated for a further 24 h. Latex extract was used as the positive control and polystyrene extract as the negative control. At the end of the incubation, the extracts were replaced by 100 μL of fresh EXPAN medium and 15 μL of 5 mg/mL MTT solution and incubated for 3.5 h. The MTT reaction was stopped by removing the MTT/EXPAN and adding 100 μL of dimethyl sulfoxide (DMSO) and stirring for 5 min. The absorbance of formazan solubilized in each well was read with a Synergy 2 microplate reader (BioTek, Winooski, VT, USA) at a wavelength of 550 nm. The measured absorbance of the samples was then normalized to the absorbance of the control cells, which had not received the extract.

### 2.8. “In Vitro” Drug Release

The release of metronidazole from the samples was investigated for 14 days by immersion in 45 mL of phosphate-buffered saline (PBS, pH 7.4, composition: 8.0 g NaCl, 1.1 g Na_2_HPO_4_, 0.2 g KCl, 0.2 g KH_2_PO_4_) at 37 °C and a rotational speed of 100 rpm. Measurement of the initial release was performed after 20 min of immersion, followed by 1, 1.8, 24, 48, 96, 168, 216, 264, and 336 h. At each interval, 4 mL of PBS was withdrawn, and the same fresh amount was replaced. In order to disregard the effects of this dilution, the following mathematical correction was made:

$$Correction\;Factor={\left(\frac{45}{45-4}\right)}^{n-1}$$

where *n* is the sequential number of the sample, 45 is the volume in milliliters of the PBS solution, and 4 is the volume in milliliters of the removed aliquot. To perform the correction, the volume of antibiotic release measured in the spectrophotometry was multiplied by this factor [19].

The amount of drug released was measured by Ultraviolet/Visible Spectroscopy (UV/Vis) equipment (mod. SP-220, Biospectro, Curitiba, Brazil). To determine the concentrations of the metronidazole in the collected solution, a standard curve of absorbance at wavelength 320 nm versus known concentrations was used.

The surface of samples before and after the drug release experiment was analyzed by SEM (Vega/Tescan, Brno, Czech Republic) microscope operating at an acceleration voltage of 15 kV in low vacuum mode).

### 2.9. Statistical Analysis

The MNZ release data were analyzed using a commercially available statistical program (SigmaPlot for Windows version 11.0). Data are reported as mean ± standard deviation. If the difference was determined to be significant after the analysis of variance, pairwise comparisons were performed using a Holm–Sidak post-hoc test, and *p* < 0.05 was considered statistically significant. The experiment was performed in triplicate with all readings for each point of the release.

## 3. Results and Discussion

### 3.1. Titanate Nanostructured Coating

The speed and quality of osseointegration are directly linked to the surface roughness and chemical composition created in the titanium. Surface treatments such as alkaline hydrothermal modifies the topography of titanium on a nanoscale [20] to generate a surface that accommodates host cells, promoting an environment conducive to cell growth and enhancing the osseointegration process [21]. Besides that, the presence of Na+ ions on the titanate surface plays an important role in the formation of apatite and therefore its bioactivity [22,23]. The alkaline hydrothermal treatment consists of submerging the sample in an alkaline solution under certain conditions of high temperature, pressure, and time to form a titanate layer with nanoscale architecture [20]. The topography resulting from this process is determined by the combination of these parameters, being attractive due to its simplicity, cost-effectiveness, and potential for large-scale manufacturing [20,24].

**Figure 1:**
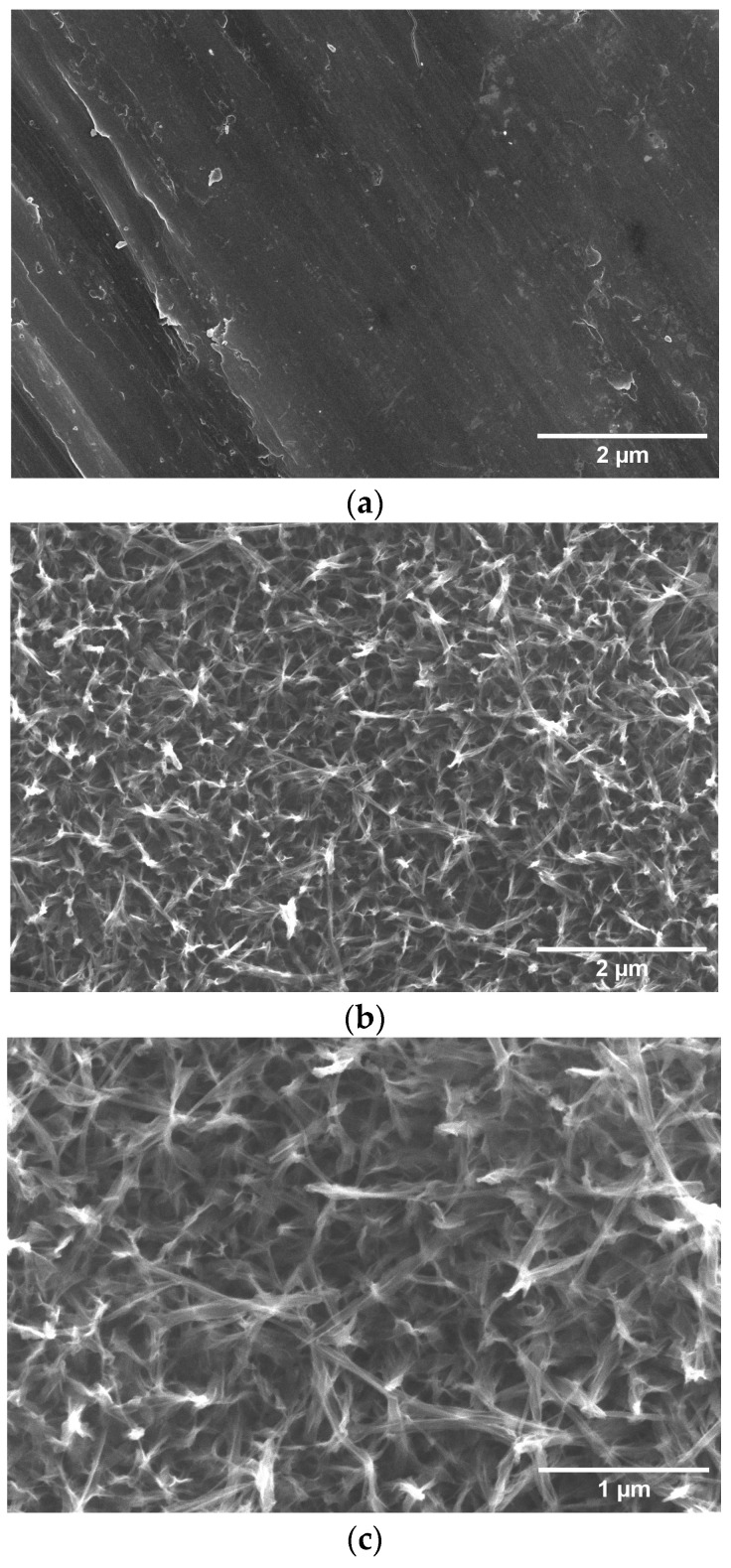
Top-view SEM image of Ti surface before (**a**) and after (**b**) alkaline hydrothermal treatment, and alkaline hydrothermal treatment at higher magnification (**c**).

Figure 1 shows a comparison of SEM images from the top view of the Ti surface before and after alkaline hydrothermal treatment under the proposed conditions (3M NaOH, 150 °C, 6 h). The hydrothermal synthesis condition chosen was based on a work by this group to be published, in which the bioactivity of different microstructures was evaluated. The Ti surface before hydrothermal treatment shows parallel grooves produced by polishing without any specific nanoscale topography (Figure 1a). On the other hand, the sample surface after treatment (Figure 1b,c) presented a microstructure composed by the intertwining of nanofibers approximately 83 nm in diameter, giving rise to micropores about 500 nm in size. This web-like morphology resembles that of the extracellular matrix of bone tissue, which can stimulate cell adhesion, proliferation, and differentiation.

Bone healing around an implant begins through cellular communication. Bone marrow mesenchymal cells interact with the implant surface, and surface properties, such as morphology, wettability, and mechanical and chemical properties, influence this process. Thus, the implant must function as a bioactive and biocompatible scaffold, with osteogenic characteristics that enable the migration, adhesion, and proliferation of cells of the osteogenic lineage [25]. The presence of a nanoscale framework that allows vascular proliferation and the passage of signaling molecules induces a desired cellular response, since interactions between cells and biomaterials occur at the nanoscale. Titanium surfaces hydrothermally treated with sodium hydroxide produce a nanoporous architecture that promotes appropriate cellular interaction with the surface, promoting the osteoblastic lineage [21].

**Figure 2:**
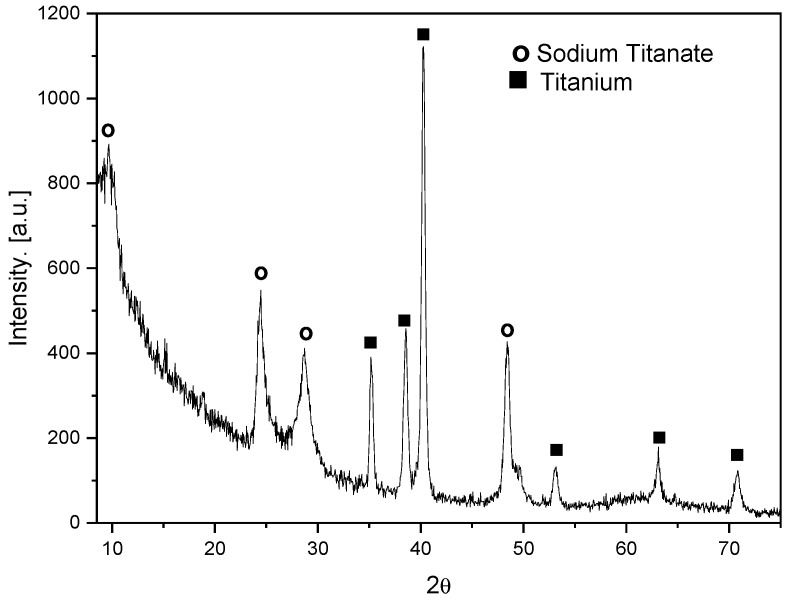
GIXRD diffractogram of Ti surface after hydrothermal treatment (TTNT).

The formation of a titanate layer was also confirmed by Grazing Incidence X-ray Diffraction (GIXRD). This is a surface sensitive technique that utilizes a small incident angle X-ray beam, which is very useful for analyzing the crystalline microstructure of thin films. Figure 2 shows the GIXRD pattern of the resulting nanostructured film, revealing typical diffraction peaks (attenuated and broadened by the nanoscale and anisotropic morphology) of layered sodium trititanate, Na_2_Ti_3_O_7_ (at 2θ = 9.6°, 24.5°, 28.7°, and 48.4°). The strong broad peak at 2θ = 9.6° can be attributed to the interlayer distance [26]. The peaks at 2θ = 35.2°, 38.6°, 40.3°, 53.2°, 63.1°, and 70.8 ° correspond to the Ti substrate.

### 3.2. “In Vitro” Bioactivity Assay

When an artificial material is implanted in a living body, it can be considered a bioactive material if it is able to connect to the living bone through a thin layer rich in calcium and phosphorus (apatite layer) without a distinct boundary. According to ISO/FDIS 23317 Standard, the formation of a bone-like apatite layer can be “in vitro” reproduced when a material is immersed in an acellular and protein-free simulated body fluid (SBF) with ion concentrations nearly equal to those of human blood plasma. Thus, under this condition, the formation of an apatite layer on the material surface is indicative of its in vivo bone-bonding ability. 

**Figure 3:**
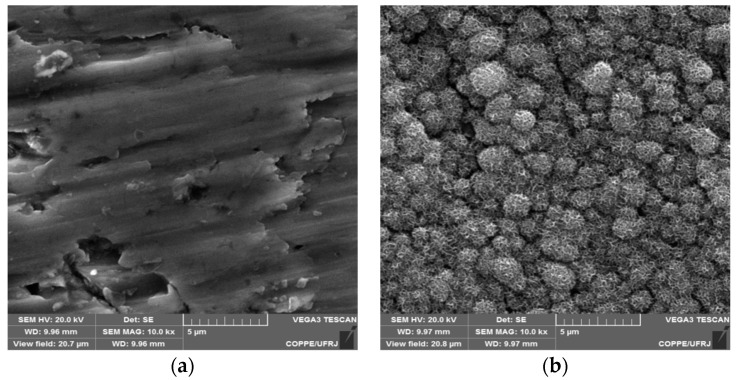
SEM images of samples’ surface after 14 days of bioactivity assaycomparing polished Ti without (**a**) and with alkaline hydrothermal treatment (**b**).

Figure 3 shows SEM images of the surface of Ti discs with (TTNT) and without hydrothermal treatment after 14 days of incubation in SBF solution (pH 7.4, 37 °C). The uniform layer of the flake-like apatite crystals can only be identified covering the sodium titanate structure (Figure 3b). The apatite formation capacity of Ti discs submitted to hydrothermal treatment can be explained by the exchange of titanate Na^+^ ions with H_3_O^+^ ions in the SBF solution to form a Ti–OH bond on the surface. In response, the surface becomes negatively charged, reacting with the calcium ions present in the SBF. These Ca^+^ ions are accumulated, making the surface positively charged and reacting with the negatively charged phosphate ions, which causes an increase in the rate of apatite nucleation [22,23].

**Figure 4:**
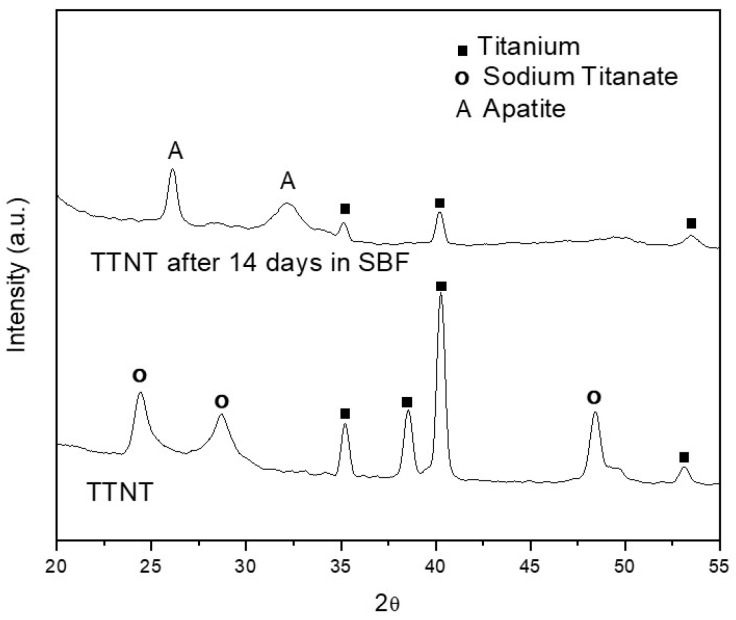
Diffractograms of Ti hydrothermally treated surface before and after immersion in SBF for 14 days.

Figure 4 shows the GIXRD diffractograms of hydrothermally treated (TTNT) Ti before and after immersion in SBF. The typical peaks of apatite at 2θ = 26° and 2θ = 32° (ICSD 9-432) were observed, and the TTNT peaks are not visible in the diffractogram of TTNT after immersion in SBF, confirming the formation of apatite’s uniform layer on the TTNT surface, thus indicating that hydrothermal treatment provides bioactivity to Ti samples.

### 3.3. Surface Wettability

Wettability influences the cascade of biological events that initiates osseointegration [27]. A hydrophilic surface results in closer contact between the titanium surface and the blood clot and cells by means of the increased availability of serum proteins with binding energy. The increase in the cell adhesion capacity by these proteins improves the adherence of the fibrin network and its retention to the surfaces of implants, as they mediate cell adhesion, followed by the cascade of coagulation and migration of undifferentiated cells and osteoblastic precursors [25,27]. 

**Figure 5:**
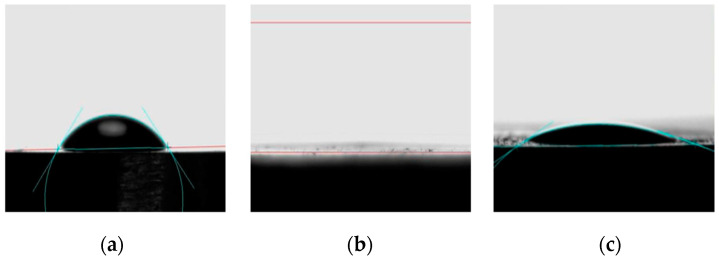
Contact angle measurements (blue lines indicate the angles). Images of a water droplet on the surface of (**a**) pure Ti surface, (**b**) Ti disc after alkaline hydrothermal treatment (TTNT), (**c**) Ti disc after addition of metronidazole (MNZ) and a layer of PVA (TTNT + MNZ + 1PVA).

Figure 5 presents images of a water droplet obtained during the contact angle measurements to evaluate the modification of surface wettability due to the surface treatment of Ti discs. In this context, the experimental group TTNT + MNZ + 1PVA was subjected to the analysis of the contact angle (Figure 5c) to monitor the change in wettability suffered after the deposition of the PVA layer when compared to the Ti pure samples (Figure 5a) and the Ti samples after hydrothermal treatment (TTNT, Figure 5c).

The contact angle of the water drop on the surface of the pure titanium samples presented the highest value (θ_c_ = 46°), indicating the less hydrophilic character of these samples. After the hydrothermal treatment, the water droplet spread out on the TTNT surface, indicating superhydrophilic behavior (θ_c_ = 0°). This could be explained considering the increase in the surface area and the formation of pores due to the presence of the titanate layer, so the decrease in contact angle may be related to the drop absorption by capillarity. Additionally, although PVA molecules present a hydrophilic character, the contact angle value increased a little after MNZ addition and PVA coating. However, the TTNT + MNZ + 1PVA samples also presented a highly hydrophilic character with θ_c_ = 22°. This fact could be explained by the smoothing of surface roughness by coating with the PVA layer, which probably hindered the water absorption by capillarity through the TTNT nanostructures. The hydrophilicity of the drug delivery system after PVA deposition for osseointegration purposes is important and indicates that this coated device could play a positive and significant role in the early stages of osseointegration.

### 3.4. “In Vitro” Metronidazole Release Evaluation

For osseointegration to occur, four stages are required: hemostasis, an inflammatory phase, a proliferative phase, and a remodeling phase. These must occur in a coordinated and organized manner [28,29], resulting in impeccable healing. The misalignment of this healing can occur in the initial inflammatory phase, which begins about 10 min after the implant is installed, creating a toxic environment. Host defense systems are activated at this stage by nonspecific molecules of bacterial origin. Polymorphonuclear leukocytes (PMN) and macrophages, and a group of glycoproteins that form membrane-perforating channels (perforins), which damage bacterial cells, are activated [30]. Therefore, the abundance of bacteria, as is the case with patients with active periodontal disease, for example, prolongs and amplifies the cellular immune response. PMNs kill bacteria through reactive radicals (oxygen species and hydroxylated groups, chlorine radicals and hypochlorite) that are also toxic to the host’s cells and to the healthy tissue around the wound. Thus, a fulminant neutrophil response can induce the loss of healthy surrounding tissues [30].

To limit the inflammatory phase, antibacterial measures are needed, such as antibiotic therapy and local disinfection. The local and controlled release of the drug directly from the surface of the implant could act to combat bacteria, preventing their adhesion, exacerbating the inflammatory process, and failing the osseointegration process. To propose a release restriction the use of PVA with different numbers of layers was suggested to compare the release in different systems.

**Figure 6:**
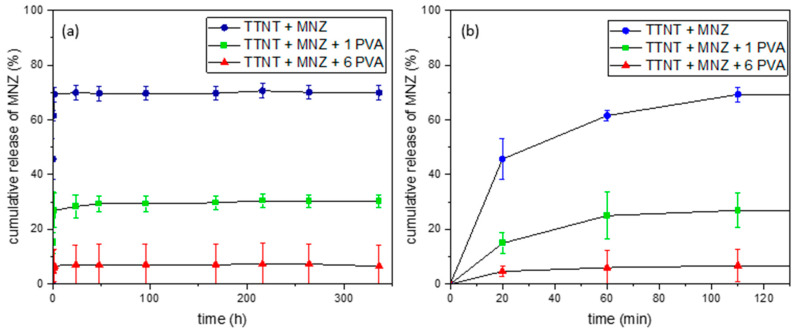
Cumulative release of MNZ with time: (**a**) whole experiment (14 days); (**b**) first 110 min.

Figure 6 shows the evolution with time of the cumulative release of metronidazole from TTNT + MNZ samples with and without PVA coating during their immersion in PBS (pH 7.4, 37 °C). Table 2 shows the accumulated percentage of MNZ released during the first 48 h.

For all groups, the total amount of MNZ was not released in the medium. The maximum release of the drug was 70%, observed for the TTNT + MNZ group. This can be justified by a possible interaction between sodium titanate and metronidazole, which would prevent these molecules from being released by diffusion during the test, a suggestion that was confirmed by FTIR analyses. The samples coated with PVA, TTNT + MNZ + 1PVA and the TTNT + MNZ + 6PVA group presented a significant reduction in the total percentage of MNZ released, at about 27% and 6%, respectively. For these groups, besides the titanate–metronidazole interaction, PVA coating acted as a physical barrier, limiting the amount of MNZ released. 

The hypothesis that there is no difference among group means was rejected by Two Way Repeated Measures ANOVA (One Factor Repetition) that assumed a statistically significant interaction between MNZ released by each experimental group and time (*p* ≤ 0.001). In Table 2, a statistical treatment with all pairwise multiple comparison procedures (Holm-Sidak method) with overall significance level = 0.05 was carried out to observe, in a more reliable way, the difference between the release times, comparing the different groups. According to the data, the TTNT + MNZ + 6PVA group reached its release constant in just 20 min, which could be justified by the efficiency of a physical barrier formed by the six layers of PVA deposited on the surface. The TTNT + MNZ + 1PVA group had its constant release in 60 min, while the TTNT + MNZ sample continued to release until 110 min. These results imply that drug release profiles can be designed according to the thickness of the PVA coating.

Although the PVA coating did not promote a gradual and prolonged release of MNZ, as expected, the metronidazole concentrations released for all groups (1.6–15.5 μg/mL) were higher than the Minimum Inhibitory Concentration (MIC) of this drug for anaerobic bacteria. MIC is the lowest antibiotic concentration required for inhibiting the growth of a specific microorganism. Previous studies [31] reported the MIC of metronidazole to eliminate anaerobic bacteria without distinction as 0.06 to 32 μg/mL. Moreover, as discussed before, the local delivery of MNZ in an immediate regime could reduce early implant complication by removing the bacterial contamination of the surgical site and, thus, avoiding disturbance in the initial inflammatory phase of wound healing, which begins 10 min after the implantation. 

### 3.5. Chemical Composition Evaluation after “In Vitro” Metronidazole Release Analysis

**Figure 7:**
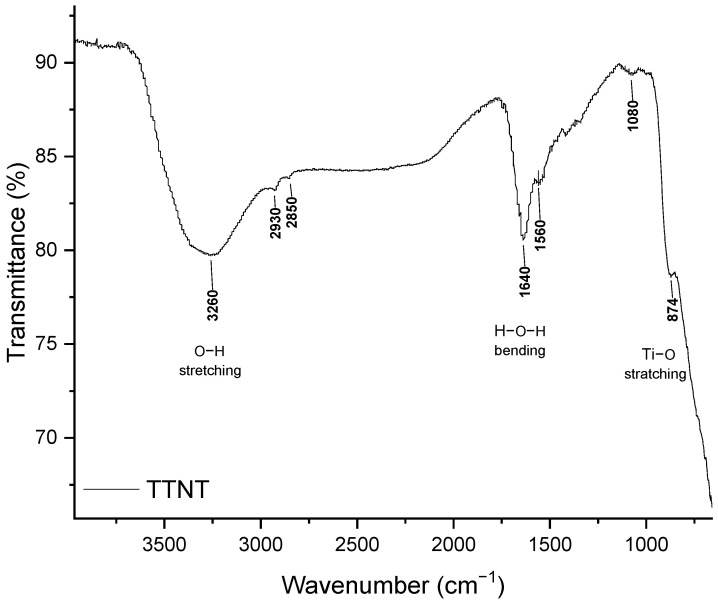
FTIR spectra of Ti sample after alkaline hydrothermal treatment (TTNT).

**Figure 8:**
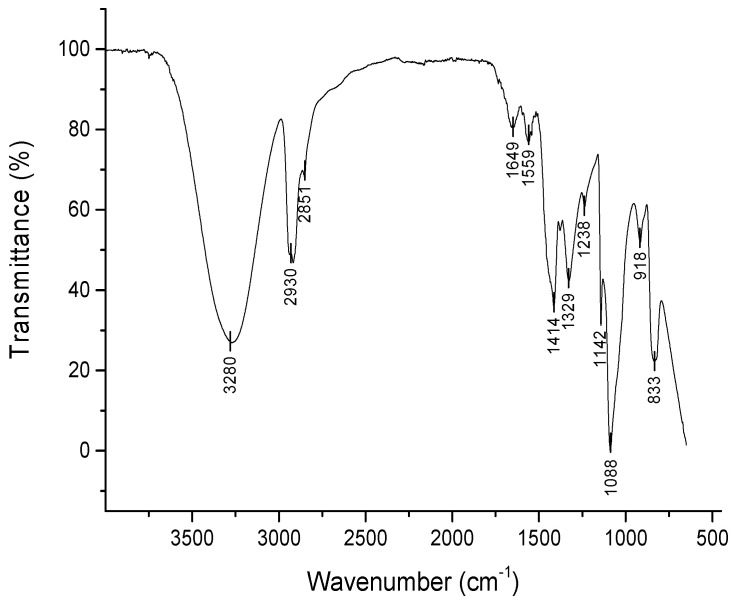
FTIR spectra of a PVA film produced by casting technique and irradiated with gamma rays at 25 KGy.

The samples were analyzed by FTIR before and after the drug release experiment. For a better interpretation of the FTIR spectra of the groups with MNZ, the FTIR spectra of nanostructured Ti samples after alkaline hydrothermal treatment (TTNT) and pure PVA film, shown in Figure 7 and Figure 8 respectively, were previously analyzed. 

In the spectrum of the TTNT sample (Figure 7), a broad band between 3000 and 3500 cm^−1^ is observed, which can be attributed to fundamental stretching vibrations in different hydroxyl groups O-H (free or linked) [2,32,33]. This may be due to the absorption of water from the atmosphere [33] and the formation of the Ti-OH bond. The band at 1630 cm^−1^ can be attributed to bending vibrations in -OH [32,33] and can indicate water absorption in the titanate when exposed to the atmosphere [2]. The set of overlapping bands in the 800 to 400 cm^−1^ range may be related to the Ti-O and Ti-O-Ti groups [32].

PVA gamma-irradiated film was used as a reference in identifying the characteristic absorption bands of the TTNT + MNZ-coated samples. The FTIR spectrum of PVA film (Figure 8) shows the following bands and their respective vibration modes: 3280 cm^−1^, hydroxyl group stretching vibration; 2930 cm^−1^ and 2851 cm^−1^, C–H stretching vibration in CH_2_ groups [34,35]; 1649 cm^−1^ and 1559 cm^−1^, C=O stretching vibration and C=C stretching vibration, respectively, of the non-hydrolyzed acetate groups [35]; 1414 cm^−1^, C–H wagging vibration in CH_2_ groups [34,35]; 1329 cm^−1^, (CH+OH) bending vibration; 1238 cm^−1^, C–C stretching vibration; 1088 cm^−1^, C–O stretching vibration; 918 cm^−1^, CH_2_ stretching vibration; 833 cm^−1^, C–C stretching vibration [35] and C–H out-of-plane vibration.

The band observed at 1142 cm^−1^ is associated with the stretching of the C–O–C bond and can be an indicator of polymer crosslinking due to gamma irradiation [17]. According to the findings of Zainuddin et al. [36], it can be suggested that alkoxy radicals were formed in the PVA chains (~CH_2_–CHO•–CH_2_~) when radiation reached the polymer. Then, these radicals underwent further transformations, leading to the formation of C–O–C bindings between mers within the same chain or between those of different chains. These crosslinking reactions in PVA during radiolysis formed a three-dimensional network of the hydrogel without the need of a chemical crosslinking agent, which could have induced cytotoxicity to the system.

**Figure 9:**
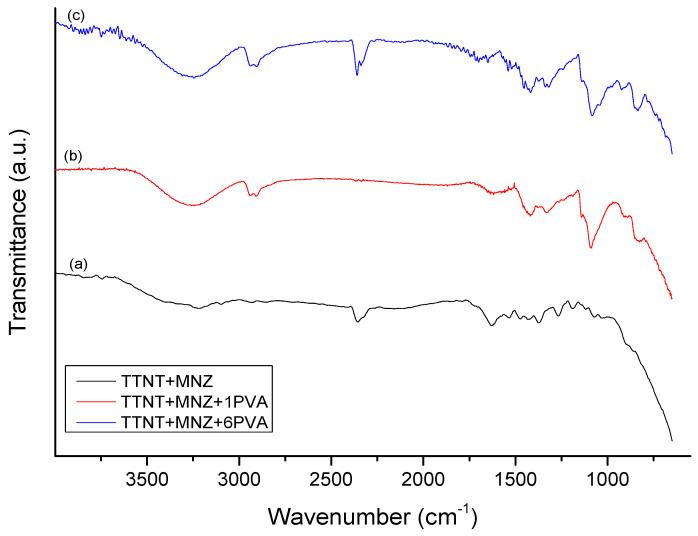
FTIR spectra of TTNT samples loaded with metronidazole: (a) TTNT + MNZ, (b) TTNT + MNZ + 1PVA and (c) TTNT + MNZ + 6PVA.

Figure 9 shows the FTIR spectra of the samples loaded with MNZ. The TTNT + MNZ spectrum is very similar to that of the samples after hydrothermal treatment (Figure 7), excepted by the presence of the absorption bands characteristic of MNZ, confirming the impregnation of MNZ in the titanate nanostructures. These MNZ bands were assigned to: anti-symmetric N-O and symmetrical elongation associated with the NO_2_ group (1533 cm^−1^ and 1371 cm^−1^, respectively); elongation N = O (1475 cm^−1^); elongation C-O (1267 cm^−1^); elongation C-N (1081 cm^−1^); OH stretching (3214 cm^−1^) [37,38]. A band at 877 cm^−1^ is also detected, which can be related both to the elongation of C-NO_2_, characteristic of MNZ, and to the elongation of Ti-O, characteristic of titanate nanostructures.

Meanwhile, absorption bands of MNZ or of titanate were not detected in the FTIR spectra of the samples coated with PVA (TTNT + MNZ + 1PVA and TTNT + MNZ + 6PVA), which suggested that the MNZ molecules and titanate nanostructure were completely covered by PVA films, corroborating the contact angle analysis. Although the spectrometer’s chamber was well purged by nitrogen, traces of gaseous carbon dioxide can be observed in some spectra. The double band at 2350 cm^−1^ presented in these spectra is assigned to asymmetric stretching modes of CO_2_ [39].

**Figure 10:**
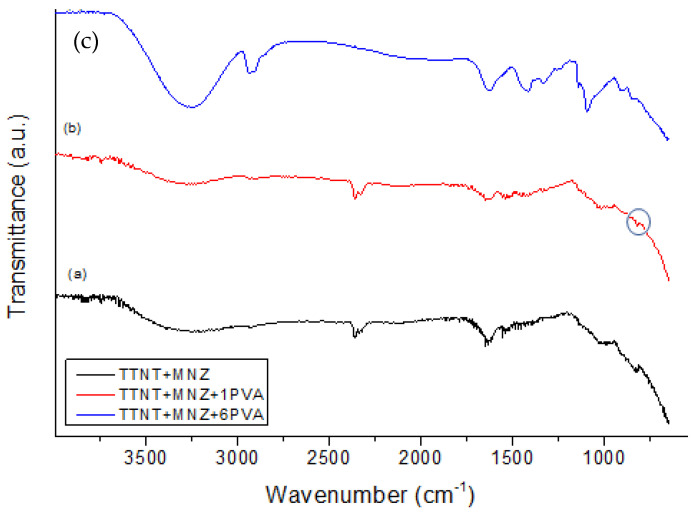
FTIR spectra of TTNT samples loaded with metronidazole, (a) TTNT + MNZ, and coated with PVA, (b) TTNT + MNZ + 1PVA and (c) TTNT + MNZ + 6PVA, after 14 days of immersion in PBS solution at 37 °C.

Figure 10 shows the FTIR spectra of samples after 14 days of immersion in PBS solution at 37 °C. In all spectra, a large band centered at about 3250 cm^−1^ (-OH stretching) and a band at 1637 cm^−1^ (H-O-H bending) can be visualized, although their intensity is higher in TTNT + MNZ + 6PVA. These can be related to the –OH group stretching vibration of PVA and also to the absorption of water from PBS solution. Characteristic bands of PVA are clearly visualized in TTNT + MNZ + 6PVA, while no characteristic vibration band of MNZ was identified. This could indicate the presence of the coating layer even after 14 days of immersion. As proposed before, probably a thick PVA coating acted as a strong physical barrier and obstructed the release of the drug. 

The spectra of TTNT + MNZ and TTNT + MNZ + 1PVA after MNZ release (Figure 10a,b) are very similar to that of pure TTNT (Figure 7), except for the presence of a small band at 1538 cm^−1^ that can be assigned to N-O antisymmetric stretching in the MNZ molecules. In the TTNT + MNZ spectrum, a shift in the band observed at 874 cm^−1^ (due to Ti-O stretching) to a lower wavenumber (835 cm^−1^), and consequently to lower energy, may be attributed to the formation of an intermolecular interaction between TTNT and MNZ in an aqueous environment. This shifted band was also observed in the TTNT + MNZ + 1PVA spectrum as a small shoulder (circled in Figure 10b). This interaction can explain the partial release of MNZ molecules from these systems, as proposed in item 3.3. The intense band at 1088 cm^−1^ related to C–O stretching vibration in the PVA molecules was not present in the TTNT + MNZ + 1PVA spectra. It can be inferred that this layer of PVA in TTNT + MNZ + 1PVA degraded in the PBS solution at least in the sample region analyzed by FTIR. 

**Figure 11:**
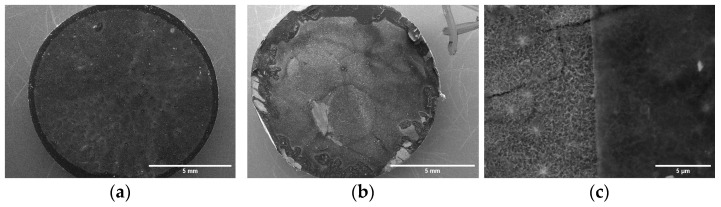
SEM images of TTNT + MNZ + 1PVA group, (**a**) before and (**b**,**c**) after “in vitro” MNZ release assay.

To confirm this assumption, SEM was used to analyze the surface of the TTNT + MNZ + 1PVA group, before and after the “in vitro” metronidazole release assay (Figure 11). An SEM image of the TTNT + MNZ + 1PVA group before the release test (Figure 11a) shows a homogeneous coating of PVA on the entire surface. The titanate nanostructure was not visualized due to the polymeric coating. The images after the MNZ release test (Figure 11b,c) reveal regions where the PVA coating has been degraded. In these regions, it is possible to observe tears in the polymeric coating and the morphology of the nanostructured film under the coating having kept intact. This partial degradation of the PVA film is in accordance with the FTIR results. Although PVA is soluble in water solution, most of its molecules remain insoluble due to crosslinking, forming a hydrogel. Thus, the much higher content of MNZ released by TTNT + MNZ + 1PVA when compared to the system with six layers of PVA may be attributed to the erosion of the coating. 

Although the thickness of the film affects its water uptake, a parallel between PVA cast film and the PVA layer of TTNT + MNZ + 1PVA can be drawn. In order to estimate the water absorption capacity of the PVA layer on the Ti surface, a PVA hydrogel was produced by the casting technique with 0.2 mm in thickness and irradiated with a dose of 25 KGy of gamma rays. This film showed a maximum absorption of 135% after 1 h of immersion in PBS pH 7.4 and reached an equilibrium swollen degree (the stage in which the hydration forces are in equilibrium with crosslinking elastic forces) of 120% after approximately 24 h. Based on this, the release of metronidazole from the TTNT + MNZ + 1PVA system may be related not only to the degradation of the coating, but also to the swelling of the PVA layer due to fluid absorption (a swelling-induced mechanism), since the period of the achievement of MNZ constant release (Figure 6, Table 2) was coincident with the point of the maximum absorption of the PVA film. 

The biological fluid uptake by systems coated with PVA provides a hydrated environment that could facilitate the distribution of the antibiotic throughout the wound surgery site and contribute to wound healing at an early stage. 

### 3.6. “In Vitro” Cytotoxicity

Considering the methodology for preparing the samples, the most critical step that could bring any damage to human cells is the hydrothermal surface treatment due to the alkaline solution used for this having the possibility of residual sodium. Moreover, although titanium implants are considered safe, with a high success rate in medical and dental applications, there are some reported cases of titanium toxicity related to corrosion, wear particle or ion release, and allergic reaction [40]. On the other hand, it can be inferred that the amount of MNZ used in our study would be safe to cells, since the exposure of human gingival fibroblasts to a dosage less than 50 μg/mL of MNZ up to 96 h did not induce the cytotoxicity effect [41]. In regard to PVA film, a previous study of the group using the same grade of PVA and employing the same methodology to produce hydrogels [17] showed that the samples were non-toxic to fibroblast cells. The irradiation method used to produce the PVA hydrogel layers leaves no residue or toxic materials in it. Furthermore, it uses only water as solvent, with no acid or toxic solvents.

In this context, the cytotoxicity of Ti and Ti + TTNT discs was assessed by evaluating human osteosarcoma MG63 cells’ response to samples after being cultured for 24 h with the extracts of the discs. The MTT colorimetric assay was employed for this proposal. This assay measures the ability of viable cells to reduce a tetrazolium salt (MTT) to formazan, a process that produces a purple color that can be measured spectrophotometrically. If the cells are healthy and viable, they will be able to reduce the MTT and produce a strong purple color. However, if the cells are damaged or dying, they will not be able to reduce the MTT and the color will be weaker or absent. 

**Figure 12:**
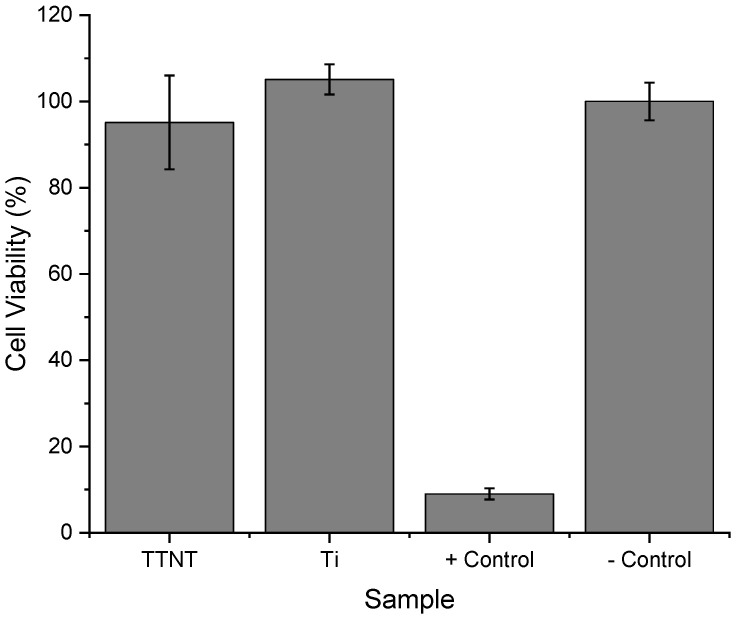
Cytotoxicity of Ti and TTNT discs extracts measured by MTT assay. + control (latex): indicates toxicity and—control (polystyrene): indicates the absence of toxicity. The results are expressed as mean ± standard deviation (SD) of six measurements for each group (n = 6).

According to Figure 12, cells cultured in extracts of the Ti and Ti + TTNT discs showed viability near the negative control (polystyrene), with no significant difference between them. These results indicate that there was no significant alteration in mitochondrial metabolic activity of cell population in the presence of the Ti and Ti + TTNT extracts, i.e., pure Ti and titanate nanostructured coating do not deliver toxic residues to the supernatant, even after 24 h soaking in a medium culture. This data is important for evaluating the safety of the biomaterial and for making decisions about its potential use in medical and dental applications.

## 4. Conclusions

In summary, alkaline hydrothermal treatment successfully produced intertwined nanostructures like a web on the titanium surface, in addition to good wettability and high bioactivity. With the aim of creating a local drug release device, the proposed surface modification strategy is simple, economical, and promising, since:

(1) It did not change the surface treatment and consequently the properties of the material;
(2) All groups reached the minimum inhibitory concentration described in the literature to help fight anaerobic bacteria with probably no cytoxicity effect;
(3) All groups allowed the immediate delivery of metronidazole, which could reduce implant complications during the early wound healing processes;
(4) Although TTNT + MNZ showed a higher percentage of antibiotic release within the studied groups, the PVA coating may absorb body fluids and water that provide distribution of MNZ throughout the wound surgery site, besides contributing to the hydration of the implant site, facilitating wound healing;
(5) The design with one layer of PVA (TTNT + MNZ + 1PVA) was shown to be the best option, since it can combine the water absorption capacity of the PVA-coated regions with the higher bioactivity of the titanate nanostructure exposed in the degraded regions of the coating. Nevertheless, the effect of the crosslinking degree of PVA on the kinetic release of metronidazole should be better investigated.

The present study serves as proof that this method of surface modification can be a new alternative to the administration of systemic drugs, combining local antibiotic therapy with a surface treatment with proven efficacy. This delivery device could become a powerful approach to improve the integration of the titanium implant with the bone in dental applications, preventing early infections and bone failure. These results encourage further studies to evaluate the biological effectiveness of the proposed surface modification methodology. 

## Figures and Tables

**Table 1:** Experimental groups.

Groups	Description
TTNT	Titanate nanostructures on Ti disc surface via hydrothermal synthesis
TTNT + MNZ	Titanate nanostructures on Ti disc surface via hydrothermal synthesis + Metronidazole
TTNT + MNZ + 1PVA	Titanate nanostructures on Ti disc surface via hydrothermal synthesis + Metronidazole + 1 layer of irradiated PVA film
TTNT + MNZ + 6PVA	Titanate nanostructures on Ti disc surface via hydrothermal synthesis + Metronidazole + 6 layers of irradiated PVA film

**Table 2:** Percentage of MNZ released in each measurement during the assay and statistical difference (different upper-case letters within a column indicate significant differences among experimental groups, different lowercase letters within a row indicate significant differences among time).

% of MNZ Released	20 Min	60 Min	110 Min	24 h	48 h
TTNT + MNZ	45.7 ^cA^	61.5 ^bA^	69.3 ^aA^	69.9 ^aA^	69.6 ^aA^
TTNT + MNZ + 1PVA	14.9 ^bB^	25.0 ^aB^	26.9 ^aB^	28.4 ^aB^	29.3 ^aB^
TTNT + MNZ + 6PVA	4.66 ^aC^	6.01 ^aC^	6.72 ^aC^	7.01 ^aC^	7.02 ^aC^

## Data Availability

The data presented in this study are available on request from the corresponding authors.
